# 5-Fluoro-3-phenyl-*N*′-(4-propyl­cyclo­hexyl­idene)-1*H*-indole-2-carbohydrazide

**DOI:** 10.1107/S1600536813018333

**Published:** 2013-07-06

**Authors:** Sevim Türktekin Çelikesir, Mehmet Akkurt, Gökçe Cihan Üstündağ, Orhan Büyükgüngör

**Affiliations:** a Department of Physics, Faculty of Sciences, Erciyes University, 38039 Kayseri, Turkey; b Department of Pharmaceutical Chemistry, Faculty of Pharmacy, Istanbul University, 34116 Beyazit, Istanbul, Turkey; c Department of Physics, Faculty of Arts and Sciences, Ondokuz Mayıs University, 55139 Samsun, Turkey

## Abstract

In the title compound, C_24_H_26_FN_3_O, the cyclo­hexane ring adopts a chair conformation; the propyl substituent is in an equatorial orientation and the bond-angle sum at the C atom bonded to the carbohydrazide N atom is 360.0°. The dihedral angle between the 1*H*-indole ring system and the phenyl ring is 82.77 (13)°. A weak intra­molecular C—H⋯π contact occurs. In the crystal, pairs of mol­ecules related by a crystallographic twofold axis are linked by bifurcated N—H⋯(O,N) hydrogen bonds; a C—H⋯O inter­action occurs between the same pair. The dimers are linked by C—H⋯F and C—H⋯π inter­actions, generating a three-dimensional network.

## Related literature

For the design and synthesis of indolylhydrazones and their cyclization products, spiro­thia­zolidinones, as potential anti­tuberculosis and anti­cancer agents, see: Akkurt *et al.* (2010 ▶, 2013 ▶); Cihan-Üstündağ & Çapan (2012 ▶). For puckering parameters, see: Cremer & Pople (1975 ▶).
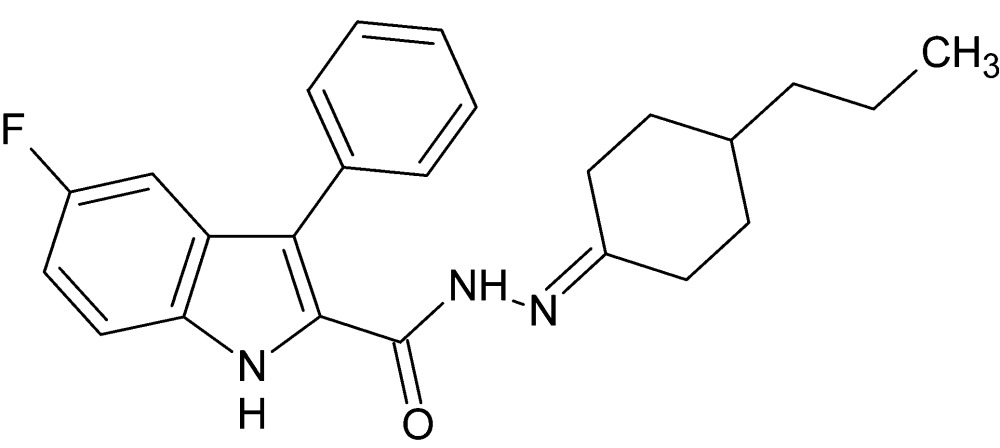


## Experimental

### 

#### Crystal data

C_24_H_26_FN_3_O
*M* *_r_* = 391.48
Tetragonal, 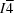

*a* = 22.6986 (11) Å
*c* = 8.4480 (5) Å
*V* = 4352.6 (5) Å^3^
*Z* = 8
Mo *K*α radiation
μ = 0.08 mm^−1^
*T* = 296 K
0.63 × 0.46 × 0.28 mm

#### Data collection

Stoe IPDS 2 diffractometer
Absorption correction: integration (Stoe & Cie, 2002 ▶) *T*_min_ = 0.957, *T*_max_ = 0.978
15640 measured reflections
4531 independent reflections
3430 reflections with *I* > 2σ(*I*)
*R*_int_ = 0.066

#### Refinement

*R*[*F* ^2^ > 2σ(*F* ^2^)] = 0.047
*wR*(*F* ^2^) = 0.092
*S* = 1.03
4531 reflections
267 parameters
2 restraints
H atoms treated by a mixture of independent and constrained refinement
Δρ_max_ = 0.13 e Å^−3^
Δρ_min_ = −0.11 e Å^−3^

### 

Data collection: *X-AREA* (Stoe & Cie, 2002 ▶); cell refinement: *X-AREA*; data reduction: *X-RED32* (Stoe & Cie, 2002 ▶); program(s) used to solve structure: *SHELXS97* (Sheldrick, 2008 ▶); program(s) used to refine structure: *SHELXL97* (Sheldrick, 2008 ▶); molecular graphics: *ORTEP-3 for Windows* (Farrugia, 2012 ▶); software used to prepare material for publication: *WinGX* (Farrugia, 2012 ▶).

## Supplementary Material

Crystal structure: contains datablock(s) global, I. DOI: 10.1107/S1600536813018333/hb7101sup1.cif

Structure factors: contains datablock(s) I. DOI: 10.1107/S1600536813018333/hb7101Isup2.hkl

Supplementary material file. DOI: 10.1107/S1600536813018333/hb7101Isup3.cml

Additional supplementary materials:  crystallographic information; 3D view; checkCIF report

## Figures and Tables

**Table 1:** Hydrogen-bond geometry (Å, °) *Cg*1, *Cg*2 and *Cg*3 are the centroids of the 1*H*-pyrrole (N1/C1/C6/C7/C14), benzene (C1–C6) and phenyl (C8–C13) rings, respectively.

*D*—H⋯*A*	*D*—H	H⋯*A*	*D*⋯*A*	*D*—H⋯*A*
N1—H1⋯O1^i^	0.86	2.50	3.048 (4)	123
N1—H1⋯N3^i^	0.86	2.32	3.170 (3)	168
C2—H2⋯O1^i^	0.93	2.51	3.112 (4)	123
C5—H5⋯F1^ii^	0.93	2.52	3.383 (3)	154
C3—H3⋯*Cg*3^iii^	0.93	2.82	3.708 (3)	160
C11—H11⋯*Cg*1^iv^	0.93	2.89	3.683 (3)	144
C17—H17*A*⋯*Cg*2^v^	0.97	2.81	3.571 (3)	136
C17—H17*B*⋯*Cg*3	0.97	2.90	3.810 (4)	157

Symmetry codes: (i) 
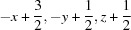
; (ii) 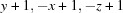; (iii) 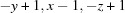; (iv) 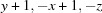; (v) 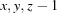.

**Fig. 1.**
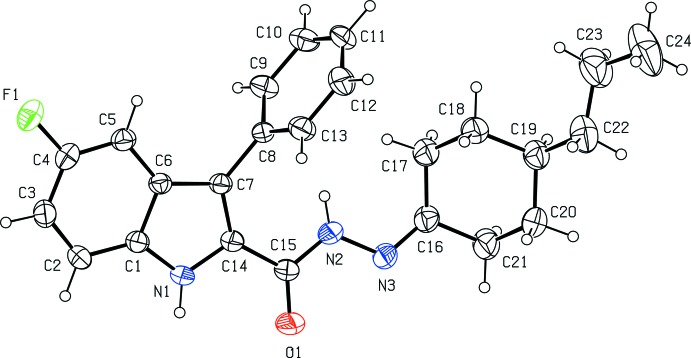
The molecular structure of (I) with displacement ellipsoids for non-H atoms drawn at the 30% probability level.

**Fig. 2.**
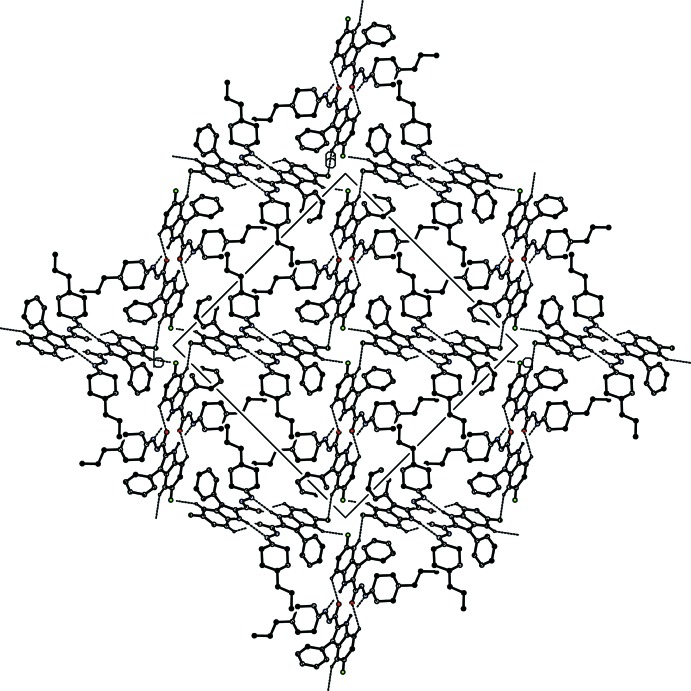
View of the packing and hydrogen bondings of the title compound, down the [001]-axis. H atoms not participating in hydrogen bonding have been omitted for clarity.

## supplementary crystallographic information

### Comment

Recently our work has been focused on the design and synthesis of novel indolylhydrazones and their cyclization products spirothiazolidinones as potential antituberculosis and anticancer agents (Akkurt *et al.*, 2010, 2013; Cihan-Üstündağ & Çapan, 2012). Within this context, we here report the synthesis and crystal structure of the title compound.

As shown in Fig. 1, the (C16–C21) cyclohexane ring of the title compound adopts a chair conformation with the puckering parameters of Q(T) = 0.540 (4) Å, θ = 176.6 (4) ° and φ = 129 (12) °. The 1*H*-indole ring system makes a dihedral angle of 82.77 (13) ° with the C8–C13 phenyl ring. The N1–C14–C15–O1, N1–C14–C15–N2, C14–C15–N2–N3, C15–N2–N3–C16, N2–N3–C16–C17 and C19–C22–C23–C24 torsion angles are 11.3 (4), -167.4 (3), 174.2 (2), 174.8 (3), -1.1 (5) and 176.7 (5) °, respectively.

In the crystal, molecules are linked by N—H···O, N—H···N, C—H···O, C—H···F hydrogen bonds, forming layers parallel to the (110) plane (Table 1, Fig. 2). In addition, C—H···π interactions also contribute to the cohesion of the crystal packing.

### Experimental

A mixture of 5-fluoro-3-phenyl-1*H*-indole-2-carbohydrazide (0.005 mol) and 4-propyl cyclohexanone (0.007 mol) was refluxed in 15 ml ethanol for 5 h. The precipitate obtained was purified by recrystallization from an ethanol-water mixture to yield colourless prisms.

Yield: 78%, mp.: 446–447.5 K. IR(KBr): υ_max_ 3366, 3243 (N—H), 1690 (C=O) cm^-1. 1^H-NMR (DMSO-d_6_/500 MHz): δ 0.78–0.88 (m and t, 4H, J= 7.3 Hz, 4-CH_2_CH_2_CH_3_, CH/CH_2_-cyc.*), 1.04–1.07 (m, 1H, CH/CH_2_-cyc.), 1.15 (br. d, 2H, J=6.3 Hz, 4-CH_2_CH_2_CH_3_-cyc.), 1.27 (br. quin, 2H, 4-CH_2_CH_2_CH_3_-cyc.), 1.47 (br. d, 2H,J=14.9 Hz, CH/CH_2_-cyc.), 1.59 (d, 1H, J=12.7 Hz, CH/CH_2_-cyc.), 1.70–1.88 (m, 2H, CH/CH_2_-cyc.), 2.13 (s, 1H, CH/CH_2_-cyc.), 2.30 (s, 1H, CH/CH_2_-cyc.), 7.12 (br. t, 2H, J=8.8 Hz, H4, H6-ind.), 7.42–7.50 (m, 6H, H7, 3-C_6_H_5_-ind.), 9.44 (s, 1H, CONH), 12.02 (s, 1H, NH) p.p.m.. ^13^C-NMR (Proton decoupled, DMSO-d_6_/100 MHz): δ 14.61 (4-CH_2_CH_2_CH_3_-cyc.), 20.05 (4-CH_2_CH_2_CH_3_-cyc.), 25.83 (CH_2_-cyc.), 32.00 (CH_2_-cyc.), 33.03 (CH_2_-cyc.), 34.43 (CH_2_-cyc.), 35.91 (CH-cyc.), 38.18 (4-CH_2_CH_2_CH_3_-cyc.), 105.02 (d, J=23.7 Hz, C4-ind.), 113.17 (d, J=26.0 Hz, C6-ind.), 114.28 (C7-ind.), 117.96 (C3-ind.), 127.36 (d, C3a-ind.), 127.89 (3-C_6_H_5_(C4)-ind.), 129.46 (3-C_6_H_5_(C3,C5)-ind.), 129.99 (C2-ind.), 130.53 (3-C_6_H_5_(C2,C6)-ind.), 132.79** (C7a-ind.), 133.72** (3-C_6_H_5_(C1)-ind.), 158.03 (C=N), 158.08 (d, J=233.6 Hz, C5-ind.), 161.94 (C=O) p.p.m.. MS (APCI+) m/*z*(%) 392 (MH^+^, 90). Analysis calculated for C_24_H_26_FN_3_O : C, 73.63; H, 6.69; N, 10.73%. Found: C, 73.43; H, 6.62; N, 10.51%.(*cyc.=cyclohexylidene, br.=broad, quin.=quintet, ind.=indole, ** interchangeable).

### Refinement

H atoms bonded to C atoms and the H atom (N1)H1 of the one of the two amide groups were positioned geometrically with C—H = 0.93 - 0.98 Å, and N—H = 0.86 Å and refined using a riding model with *U*_iso_(H) = 1.2 or 1.5*U*_eq_(C,*N*). The H atom (N2)H2A of the second groups were found in a difference Fourier map, restrained with N—H = 0.86 (2) Å and refined with *U*_iso_ = 1.2*U*_eq_(N). The absolute structure was indeterminate in the present experiment.

### Crystal data

**Table:**

C_24_H_26_FN_3_O	*D*_x_ = 1.195 Mg m^−^^3^
*M**_r_* = 391.48	Mo *K*α radiation, λ = 0.71073 Å
Tetragonal, *I*4	Cell parameters from 20884 reflections
Hall symbol: I -4	θ = 2.5–27.4°
*a* = 22.6986 (11) Å	µ = 0.08 mm^−^^1^
*c* = 8.4480 (5) Å	*T* = 296 K
*V* = 4352.6 (5) Å^3^	Prism, colourless
*Z* = 8	0.63 × 0.46 × 0.28 mm
*F*(000) = 1664	

### Data collection

**Table:**

Stoe IPDS 2 diffractometer	4531 independent reflections
Radiation source: sealed X-ray tube, 12 x 0.4 mm long-fine focus	3430 reflections with *I* > 2σ(*I*)
Plane graphite monochromator	*R*_int_ = 0.066
Detector resolution: 6.67 pixels mm^-1^	θ_max_ = 26.5°, θ_min_ = 2.5°
ω–scans	*h* = −28→27
Absorption correction: integration (Stoe & Cie, 2002)	*k* = −28→28
*T*_min_ = 0.957, *T*_max_ = 0.978	*l* = −10→10
15640 measured reflections	

### Refinement

**Table:**

Refinement on *F*^2^	2 restraints
Least-squares matrix: full	Hydrogen site location: mixed
*R*[*F*^2^ > 2σ(*F*^2^)] = 0.047	H atoms treated by a mixture of independent and constrained refinement
*wR*(*F*^2^) = 0.092	W = 1/[Σ^2^(*F*O^2^) + (0.0396*P*)^2^ + 0.3772*P*] WHERE *P* = (*F*O^2^ + 2*F*C^2^)/3
*S* = 1.03	(Δ/σ)_max_ < 0.001
4531 reflections	Δρ_max_ = 0.13 e Å^−^^3^
267 parameters	Δρ_min_ = −0.11 e Å^−^^3^

### Special details

**Table:**

Geometry. Bond distances, angles *etc*. have been calculated using the rounded fractional coordinates. All su's are estimated from the variances of the (full) variance-covariance matrix. The cell e.s.d.'s are taken into account in the estimation of distances, angles and torsion angles
Refinement. Refinement on *F*^2^ for ALL reflections except those flagged by the user for potential systematic errors. Weighted *R*-factors *wR* and all goodnesses of fit *S* are based on *F*^2^, conventional *R*-factors *R* are based on *F*, with *F* set to zero for negative *F*^2^. The observed criterion of *F*^2^ > σ(*F*^2^) is used only for calculating -*R*-factor-obs *etc*. and is not relevant to the choice of reflections for refinement. *R*-factors based on *F*^2^ are statistically about twice as large as those based on *F*, and *R*-factors based on ALL data will be even larger.

### Fractional atomic coordinates and isotropic or equivalent isotropic displacement parameters (Å^2^)

**Table:**

	*x*	*y*	*z*	*U*_iso_*/*U*_eq_	
F1	0.95641 (9)	0.05822 (8)	0.6774 (3)	0.0742 (7)	
O1	0.77085 (10)	0.26762 (14)	0.0944 (4)	0.1013 (11)	
N1	0.80598 (10)	0.18855 (10)	0.3230 (3)	0.0513 (8)	
N2	0.84810 (11)	0.24602 (12)	−0.0590 (3)	0.0588 (9)	
N3	0.83121 (11)	0.28546 (12)	−0.1750 (3)	0.0608 (9)	
C1	0.83656 (11)	0.15385 (12)	0.4270 (4)	0.0476 (9)	
C2	0.82036 (14)	0.13032 (13)	0.5733 (4)	0.0587 (10)	
C3	0.86172 (14)	0.09835 (13)	0.6542 (4)	0.0605 (11)	
C4	0.91761 (14)	0.09077 (12)	0.5911 (4)	0.0559 (10)	
C5	0.93519 (12)	0.11312 (12)	0.4498 (4)	0.0507 (9)	
C6	0.89358 (11)	0.14621 (11)	0.3643 (3)	0.0432 (8)	
C7	0.89623 (11)	0.17781 (11)	0.2192 (3)	0.0428 (8)	
C8	0.94986 (11)	0.18498 (11)	0.1185 (3)	0.0443 (9)	
C9	0.96716 (13)	0.14270 (14)	0.0110 (4)	0.0584 (11)	
C10	1.01697 (14)	0.15077 (16)	−0.0816 (4)	0.0665 (11)	
C11	1.05025 (13)	0.20044 (16)	−0.0662 (4)	0.0667 (11)	
C12	1.03435 (15)	0.24189 (17)	0.0407 (5)	0.0777 (14)	
C13	0.98449 (13)	0.23467 (14)	0.1336 (4)	0.0634 (11)	
C14	0.84140 (11)	0.20304 (11)	0.1986 (3)	0.0465 (9)	
C15	0.81674 (12)	0.24211 (14)	0.0752 (4)	0.0566 (10)	
C16	0.86492 (14)	0.29068 (14)	−0.2948 (4)	0.0592 (11)	
C17	0.92192 (15)	0.26047 (14)	−0.3283 (4)	0.0687 (11)	
C18	0.97104 (15)	0.30538 (16)	−0.3529 (4)	0.0723 (12)	
C19	0.95600 (16)	0.35201 (15)	−0.4750 (4)	0.0673 (11)	
C20	0.89727 (17)	0.38012 (17)	−0.4386 (5)	0.0780 (12)	
C21	0.84788 (16)	0.33486 (19)	−0.4175 (5)	0.0830 (14)	
C22	1.0046 (2)	0.3974 (2)	−0.4948 (5)	0.0937 (16)	
C23	1.0627 (2)	0.3747 (3)	−0.5558 (8)	0.131 (3)	
C24	1.1078 (3)	0.4215 (4)	−0.5818 (8)	0.169 (4)	
H1	0.77000	0.19950	0.33440	0.0620*	
H2	0.78280	0.13620	0.61440	0.0700*	
H2A	0.8751 (11)	0.2227 (12)	−0.060 (3)	0.060 (9)*	
H3	0.85230	0.08170	0.75150	0.0730*	
H5	0.97300	0.10680	0.41120	0.0610*	
H9	0.94520	0.10830	0.00050	0.0700*	
H10	1.02770	0.12210	−0.15470	0.0800*	
H11	1.08360	0.20580	−0.12860	0.0800*	
H12	1.05710	0.27570	0.05200	0.0930*	
H13	0.97430	0.26350	0.20680	0.0760*	
H17A	0.91790	0.23630	−0.42240	0.0830*	
H17B	0.93190	0.23480	−0.24040	0.0830*	
H18A	0.97950	0.32450	−0.25280	0.0870*	
H18B	1.00640	0.28490	−0.38620	0.0870*	
H19	0.95180	0.33180	−0.57680	0.0810*	
H20A	0.88690	0.40670	−0.52400	0.0930*	
H20B	0.90080	0.40320	−0.34250	0.0930*	
H21A	0.81190	0.35470	−0.38560	0.1000*	
H21B	0.84050	0.31500	−0.51730	0.1000*	
H22A	0.99070	0.42770	−0.56670	0.1130*	
H22B	1.01140	0.41600	−0.39310	0.1130*	
H23A	1.07820	0.34630	−0.48090	0.1570*	
H23B	1.05600	0.35430	−0.65500	0.1570*	
H24A	1.11610	0.44090	−0.48330	0.2030*	
H24B	1.09310	0.44970	−0.65680	0.2030*	
H24C	1.14330	0.40400	−0.62200	0.2030*	

### Atomic displacement parameters (Å^2^)

**Table:**

	*U*^11^	*U*^22^	*U*^33^	*U*^12^	*U*^13^	*U*^23^
F1	0.0820 (13)	0.0652 (11)	0.0755 (13)	0.0059 (9)	−0.0167 (10)	0.0183 (10)
O1	0.0639 (15)	0.135 (2)	0.105 (2)	0.0507 (15)	0.0263 (15)	0.0539 (19)
N1	0.0347 (11)	0.0569 (14)	0.0623 (15)	0.0076 (10)	0.0116 (11)	0.0062 (12)
N2	0.0488 (14)	0.0670 (16)	0.0605 (16)	0.0165 (13)	−0.0026 (13)	0.0149 (14)
N3	0.0486 (13)	0.0740 (17)	0.0599 (16)	0.0073 (12)	−0.0061 (14)	0.0195 (14)
C1	0.0434 (14)	0.0435 (14)	0.0558 (17)	0.0010 (11)	0.0067 (14)	−0.0004 (14)
C2	0.0553 (17)	0.0578 (17)	0.0630 (19)	0.0022 (14)	0.0196 (16)	0.0060 (16)
C3	0.074 (2)	0.0535 (17)	0.0540 (18)	−0.0014 (15)	0.0104 (17)	0.0073 (15)
C4	0.0631 (19)	0.0438 (16)	0.0609 (19)	0.0019 (13)	−0.0076 (16)	0.0050 (15)
C5	0.0426 (14)	0.0480 (15)	0.0616 (18)	0.0028 (12)	−0.0002 (14)	0.0011 (14)
C6	0.0405 (14)	0.0390 (13)	0.0502 (15)	0.0013 (11)	0.0035 (12)	−0.0029 (12)
C7	0.0364 (13)	0.0437 (14)	0.0483 (16)	0.0026 (11)	0.0025 (12)	−0.0005 (12)
C8	0.0336 (13)	0.0507 (15)	0.0487 (17)	0.0053 (11)	−0.0003 (12)	0.0087 (13)
C9	0.0444 (16)	0.0658 (19)	0.065 (2)	−0.0009 (14)	0.0054 (15)	−0.0084 (16)
C10	0.0536 (18)	0.084 (2)	0.062 (2)	0.0113 (16)	0.0098 (16)	−0.0081 (18)
C11	0.0432 (16)	0.087 (2)	0.070 (2)	0.0038 (16)	0.0174 (16)	0.014 (2)
C12	0.060 (2)	0.066 (2)	0.107 (3)	−0.0134 (17)	0.020 (2)	0.013 (2)
C13	0.0551 (18)	0.0540 (17)	0.081 (2)	−0.0016 (14)	0.0174 (17)	−0.0034 (16)
C14	0.0390 (14)	0.0482 (15)	0.0523 (16)	0.0023 (12)	0.0038 (13)	0.0020 (13)
C15	0.0379 (14)	0.0648 (18)	0.067 (2)	0.0075 (13)	0.0065 (15)	0.0128 (16)
C16	0.0519 (17)	0.0660 (19)	0.0596 (19)	−0.0019 (15)	−0.0097 (16)	0.0107 (16)
C17	0.078 (2)	0.0631 (19)	0.065 (2)	0.0053 (16)	0.0121 (18)	0.0046 (18)
C18	0.060 (2)	0.088 (2)	0.069 (2)	0.0106 (17)	0.0040 (18)	0.015 (2)
C19	0.070 (2)	0.075 (2)	0.0570 (19)	−0.0035 (17)	0.0010 (16)	0.0060 (17)
C20	0.085 (2)	0.079 (2)	0.070 (2)	0.0109 (19)	0.002 (2)	0.024 (2)
C21	0.064 (2)	0.109 (3)	0.076 (2)	0.0032 (19)	−0.0066 (19)	0.034 (2)
C22	0.095 (3)	0.109 (3)	0.077 (2)	−0.022 (2)	−0.005 (2)	0.022 (2)
C23	0.084 (3)	0.169 (5)	0.139 (5)	−0.026 (3)	0.007 (3)	0.047 (4)
C24	0.113 (4)	0.272 (9)	0.122 (5)	−0.080 (5)	−0.013 (4)	0.074 (5)

### Geometric parameters (Å, º)

**Table:**

F1—C4	1.361 (4)	C19—C22	1.519 (6)
O1—C15	1.203 (4)	C19—C20	1.510 (5)
N1—C1	1.369 (4)	C20—C21	1.531 (6)
N1—C14	1.364 (4)	C22—C23	1.507 (7)
N2—N3	1.382 (4)	C23—C24	1.492 (10)
N2—C15	1.342 (4)	C2—H2	0.9300
N3—C16	1.274 (4)	C3—H3	0.9300
N1—H1	0.8600	C5—H5	0.9300
N2—H2A	0.81 (3)	C9—H9	0.9300
C1—C2	1.396 (5)	C10—H10	0.9300
C1—C6	1.409 (4)	C11—H11	0.9300
C2—C3	1.369 (4)	C12—H12	0.9300
C3—C4	1.387 (5)	C13—H13	0.9300
C4—C5	1.357 (5)	C17—H17A	0.9700
C5—C6	1.406 (4)	C17—H17B	0.9700
C6—C7	1.422 (4)	C18—H18A	0.9700
C7—C8	1.494 (4)	C18—H18B	0.9700
C7—C14	1.381 (4)	C19—H19	0.9800
C8—C9	1.378 (4)	C20—H20A	0.9700
C8—C13	1.381 (4)	C20—H20B	0.9700
C9—C10	1.387 (4)	C21—H21A	0.9700
C10—C11	1.363 (5)	C21—H21B	0.9700
C11—C12	1.353 (5)	C22—H22A	0.9700
C12—C13	1.387 (5)	C22—H22B	0.9700
C14—C15	1.479 (4)	C23—H23A	0.9700
C16—C21	1.493 (5)	C23—H23B	0.9700
C16—C17	1.491 (5)	C24—H24A	0.9600
C17—C18	1.525 (5)	C24—H24B	0.9600
C18—C19	1.517 (5)	C24—H24C	0.9600
			
C1—N1—C14	109.5 (2)	C4—C5—H5	122.00
N3—N2—C15	119.7 (3)	C6—C5—H5	121.00
N2—N3—C16	117.2 (3)	C8—C9—H9	120.00
C1—N1—H1	125.00	C10—C9—H9	120.00
C14—N1—H1	125.00	C9—C10—H10	120.00
N3—N2—H2A	128.8 (18)	C11—C10—H10	120.00
C15—N2—H2A	111.5 (18)	C10—C11—H11	120.00
N1—C1—C6	107.2 (3)	C12—C11—H11	120.00
C2—C1—C6	121.9 (3)	C11—C12—H12	120.00
N1—C1—C2	130.9 (3)	C13—C12—H12	120.00
C1—C2—C3	117.7 (3)	C8—C13—H13	120.00
C2—C3—C4	120.1 (3)	C12—C13—H13	120.00
F1—C4—C3	117.0 (3)	C16—C17—H17A	110.00
C3—C4—C5	124.1 (3)	C16—C17—H17B	109.00
F1—C4—C5	118.9 (3)	C18—C17—H17A	110.00
C4—C5—C6	117.0 (3)	C18—C17—H17B	110.00
C1—C6—C5	119.3 (3)	H17A—C17—H17B	108.00
C5—C6—C7	133.2 (2)	C17—C18—H18A	109.00
C1—C6—C7	107.5 (2)	C17—C18—H18B	109.00
C6—C7—C14	106.3 (2)	C19—C18—H18A	109.00
C8—C7—C14	128.1 (2)	C19—C18—H18B	109.00
C6—C7—C8	125.5 (2)	H18A—C18—H18B	108.00
C7—C8—C13	120.0 (2)	C18—C19—H19	107.00
C9—C8—C13	117.9 (3)	C20—C19—H19	107.00
C7—C8—C9	122.1 (2)	C22—C19—H19	107.00
C8—C9—C10	120.8 (3)	C19—C20—H20A	109.00
C9—C10—C11	120.5 (3)	C19—C20—H20B	109.00
C10—C11—C12	119.4 (3)	C21—C20—H20A	109.00
C11—C12—C13	120.9 (3)	C21—C20—H20B	109.00
C8—C13—C12	120.6 (3)	H20A—C20—H20B	108.00
N1—C14—C15	117.7 (2)	C16—C21—H21A	110.00
C7—C14—C15	132.7 (2)	C16—C21—H21B	110.00
N1—C14—C7	109.5 (2)	C20—C21—H21A	110.00
N2—C15—C14	115.8 (2)	C20—C21—H21B	110.00
O1—C15—N2	122.8 (3)	H21A—C21—H21B	108.00
O1—C15—C14	121.4 (3)	C19—C22—H22A	108.00
N3—C16—C21	117.3 (3)	C19—C22—H22B	108.00
C17—C16—C21	113.7 (3)	C23—C22—H22A	108.00
N3—C16—C17	129.0 (3)	C23—C22—H22B	108.00
C16—C17—C18	110.7 (3)	H22A—C22—H22B	107.00
C17—C18—C19	113.2 (3)	C22—C23—H23A	109.00
C18—C19—C20	110.8 (3)	C22—C23—H23B	109.00
C18—C19—C22	112.6 (3)	C24—C23—H23A	109.00
C20—C19—C22	112.2 (3)	C24—C23—H23B	109.00
C19—C20—C21	112.8 (3)	H23A—C23—H23B	108.00
C16—C21—C20	110.0 (3)	C23—C24—H24A	109.00
C19—C22—C23	116.2 (4)	C23—C24—H24B	109.00
C22—C23—C24	114.1 (6)	C23—C24—H24C	109.00
C1—C2—H2	121.00	H24A—C24—H24B	110.00
C3—C2—H2	121.00	H24A—C24—H24C	109.00
C2—C3—H3	120.00	H24B—C24—H24C	110.00
C4—C3—H3	120.00		
			
C1—N1—C14—C15	−178.2 (2)	C14—C7—C8—C13	79.6 (4)
C14—N1—C1—C2	176.9 (3)	C14—C7—C8—C9	−101.6 (3)
C14—N1—C1—C6	−0.2 (3)	C8—C7—C14—C15	2.2 (5)
C1—N1—C14—C7	0.0 (3)	C6—C7—C8—C13	−95.4 (3)
C15—N2—N3—C16	174.8 (3)	C9—C8—C13—C12	1.3 (5)
N3—N2—C15—O1	7.1 (5)	C7—C8—C13—C12	−179.9 (3)
N3—N2—C15—C14	−174.2 (2)	C7—C8—C9—C10	179.6 (3)
N2—N3—C16—C21	−177.8 (3)	C13—C8—C9—C10	−1.6 (4)
N2—N3—C16—C17	−1.1 (5)	C8—C9—C10—C11	1.0 (5)
C2—C1—C6—C7	−177.2 (3)	C9—C10—C11—C12	0.2 (5)
N1—C1—C2—C3	−177.8 (3)	C10—C11—C12—C13	−0.5 (5)
C6—C1—C2—C3	−1.0 (4)	C11—C12—C13—C8	−0.2 (5)
N1—C1—C6—C7	0.3 (3)	N1—C14—C15—N2	−167.4 (3)
C2—C1—C6—C5	1.1 (4)	C7—C14—C15—O1	−166.4 (3)
N1—C1—C6—C5	178.6 (2)	C7—C14—C15—N2	14.9 (5)
C1—C2—C3—C4	0.6 (4)	N1—C14—C15—O1	11.3 (4)
C2—C3—C4—F1	−179.6 (3)	N3—C16—C17—C18	−122.2 (4)
C2—C3—C4—C5	−0.3 (5)	C21—C16—C17—C18	54.6 (4)
C3—C4—C5—C6	0.4 (4)	N3—C16—C21—C20	121.6 (3)
F1—C4—C5—C6	179.7 (2)	C17—C16—C21—C20	−55.6 (4)
C4—C5—C6—C7	177.0 (3)	C16—C17—C18—C19	−52.3 (4)
C4—C5—C6—C1	−0.8 (4)	C17—C18—C19—C20	51.8 (4)
C5—C6—C7—C8	−2.3 (5)	C17—C18—C19—C22	178.4 (3)
C1—C6—C7—C14	−0.2 (3)	C18—C19—C20—C21	−52.9 (4)
C5—C6—C7—C14	−178.2 (3)	C22—C19—C20—C21	−179.7 (3)
C1—C6—C7—C8	175.7 (2)	C18—C19—C22—C23	63.2 (5)
C6—C7—C14—N1	0.1 (3)	C20—C19—C22—C23	−170.9 (4)
C6—C7—C8—C9	83.4 (4)	C19—C20—C21—C16	54.5 (4)
C6—C7—C14—C15	178.0 (3)	C19—C22—C23—C24	176.7 (5)
C8—C7—C14—N1	−175.6 (2)		

### Hydrogen-bond geometry (Å, º)

Cg1, Cg2 and Cg3 are the centroids of the 1*H*-pyrrole (N1/C1/C6/C7/C14), benzene (C1–C6) and phenyl (C8–C13) rings, respectively.

**Table:**

*D*—H···*A*	*D*—H	H···*A*	*D*···*A*	*D*—H···*A*
N1—H1···O1^i^	0.86	2.50	3.048 (4)	123
N1—H1···N3^i^	0.86	2.32	3.170 (3)	168
C2—H2···O1^i^	0.93	2.51	3.112 (4)	123
C5—H5···F1^ii^	0.93	2.52	3.383 (3)	154
C3—H3···*Cg*3^iii^	0.93	2.82	3.708 (3)	160
C11—H11···*Cg*1^iv^	0.93	2.89	3.683 (3)	144
C17—H17*A*···*Cg*2^v^	0.97	2.81	3.571 (3)	136
C17—H17*B*···*Cg*3	0.97	2.90	3.810 (4)	157

Symmetry codes: (i) −*x*+3/2, −*y*+1/2, *z*+1/2; (ii) *y*+1, −*x*+1, −*z*+1; (iii) −*y*+1, *x*−1, −*z*+1; (iv) *y*+1, −*x*+1, −*z*; (v) *x*, *y*, *z*−1.
